# Co-creation and self-evaluation: An accountability mechanism process in water, sanitation and hygiene services delivery in childcare centres in Nairobi's informal settlements

**DOI:** 10.3389/fpubh.2022.1035284

**Published:** 2023-01-12

**Authors:** Ivy Chumo, Caroline Kabaria, Helen Elsey, Kim Ozano, Penelope A. Phillips-Howard, Blessing Mberu

**Affiliations:** ^1^Urbanization and Wellbeing Unit, African Population and Health Research Center (APHRC), Nairobi, Kenya; ^2^Hull York Medical School, University of York, York, United Kingdom; ^3^Department of Clinical Sciences, Liverpool School of Tropical Medicine (LSTM), Institute of Public Health, Liverpool, United Kingdom

**Keywords:** accountability, childcare centers, water sanitation and hygiene (WASH), co-creation and co-production, children, urban governance, urban health, informal settlements

## Abstract

**Background:**

Accountability strategies are expected to enhance access to water, sanitation and hygiene (WASH) service delivery in low-and middle-income countries (LMIC). Conventional formal social accountability mechanisms (SAMs) for WASH service delivery have been inadequate to meet the needs of residents in informal settlements in LMICs. This has prompted growing interest in alternative informal SAMs (iSAMs) in Nairobi's informal settlements. To date, iSAMs have shown a limited effect, often due to implementation failures and poor contextual fit. In childcare centers in Nairobi's informal settlements, co-creation of the iSAMs process, where parents, childcare managers, researchers and other WASH stakeholders, contribute to the design and implementation of iSAMs, is an approach with the potential to meet urgent WASH needs. However, to our knowledge, no study has documented (1) co-creating iSAMs processes for WASH service delivery in childcare centers and (2) self-evaluation of the co-creation process in the informal settlements.

**Methods:**

We used a qualitative approach where we collected data through workshops and focus group discussions to document and inform (a) co-creation processes of SAMs for WASH service delivery in childcare centers and (b) self-evaluation of the co-creation process. We used a framework approach for data analysis informed by Coleman's framework.

**Results:**

Study participants co-created an iSAM process that entailed: definition; action and sharing information; judging and assessing; and learning and adapting iSAMs. The four steps were considered to increase the capability to meet WASH needs in childcare centers. We also documented a self-evaluation appraisal of the iSAM process. Study participants described that the co-creation process could improve understanding, inclusion, ownership and performance in WASH service delivery. Negative appraisals described included financial, structural, social and time constraints.

**Conclusion:**

We conclude that the co-creation process could address contextual barriers which are often overlooked, as it allows understanding of issues through the ‘eyes' of people who experience service delivery issues. Further, we conclude that sustainable and equitable WASH service delivery in childcare centers in informal settlements needs research that goes beyond raising awareness to fully engage and co-create to ensure that novel solutions are developed at an appropriate scale to meet specific needs. We recommend that actors should incorporate co-creation in identification of feasible structures for WASH service delivery in childcare centers and other contexts.

## Introduction

Childcare centers are common particularly in informal settlements in low and middle-income countries (LMICs), due to an increased number of working hours in rapidly expanding urban areas (1). Childcare centers have a growing significance in shaping life course trajectories for children (1, 2). A large gulf has opened up between the transformative promises offered by policy actors and the insufficient, often low quality and inequitable realities of access to water, sanitation, and hygiene (WASH) services by children under 5 years old in childcare centers in LMIC (2). Many of the basic services are neither statutory nor compulsory, with limitations of finance, governance and a growing reliance on non-governmental organization (NGO), faith-based and private-for-profit led initiatives (3, 4). As such, children under 5 years of age are among the marginalized and under-represented in access to services, including WASH service delivery in childcare centers (4, 5), more so in informal settlements (6, 7).

Access to WASH services in childcare centers is important for child health and wellbeing (8). Improved WASH practices are essential for reduced transmissions of WASH-related diseases (9, 10). Diseases such as diarrhea, parasitic worm infections, skin and eye diseases, need to be tackled by implementing guidelines that facilitate WASH service delivery in settings including childcare centers in informal settlements (3, 6). Implementation of the guidelines includes service providers observing WASH standards and indicators (9, 11). When the service providers are responsible for appropriate WASH practices, accountability mechanisms can play a significant role in ensuring good practices are upheld (7). Social accountability is a process in which individuals are obliged to explain their actions to other individuals, who have the right to judge them and to administer positive or negative consequences in response to the actions taken (12). Improved WASH practices require social accountability to protect young children's right to adequate WASH services guaranteed in the Convention on the Rights of the Child (13). Social accountability mechanisms (SAMs) may be one way to improve access to WASH services (12, 14). Yet, the failure of conventional formal accountability mechanisms in WASH service delivery has prompted growing interest in alternative models of informal SAMs (15, 16). Informal SAMs (iSAMs) for improving WASH service delivery have shown a limited effect (17, 18), because the iSAMs processes are usually not carried out as intended, as such the implementation fidelity is low (19, 20), due to the poor contextual fit of the processes (20–22) and a lack of readiness for change (20). To develop a more efficient and responsive process, co-creation, where childcare managers, parents and relevant actors develop the iSAM process is increasingly encouraged (21).

Co-creation refers to a collective creativity that is experienced and performed jointly by a group of people (19, 20), where end-users collaborate with service providers and other non-academic stakeholders such as policymakers, and managers (23, 24). Co-creating iSAMs enhance contextual fit (20), which in this study, involves tailoring iSAMs to the childcare context for the approaches to be responsive to the WASH service needs of children in informal settlements (16, 22). Co-creation also enhances readiness for change, which is vital for behavior change for the iSAMs to be successfully implemented (22, 25). Instead of only relying on theory to guide the creation of approaches, local knowledge regarding structures and values must be utilized collaboratively (20, 23). The collaboration can include the development of an agenda, design and/or implementation (23), with the implementation process often not considered (26). Although many benefits of involving key actors in developing implementation approaches have been proposed (25), few studies have explored co-creation processes concerning iSAMs (27, 28) and evaluation of the co-creation process. Therefore, this study seeks to document a co-creation processes for WASH service delivery in childcare centers and a self-evaluation of the co-creation process. In addressing these objectives we employed qualitative and participatory study methodologies of workshops and focus group discussions to answer the following questions: (1) how does the co-creation process of iSAMs operate in WASH service delivery in childcare centers? and (2) how do stakeholders self-evaluate the co-creation process?

## Conceptual framework

**Figure 1:**
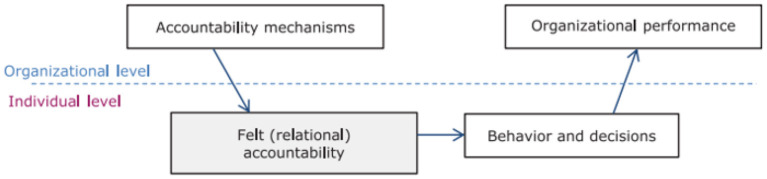
Conceptual framework (adapted from Coleman's boat applied to accountability).

Co-creation requires collaboration and capital that is notably key in WASH service delivery in childcare centres. As such, this study is grounded on Coleman's foundations of social framework, with a focus on human, physical and social capital, and their interactions. Specifically, the aim of Coleman's concept of social capital is to apply the economists' principle of rational action in the analysis of social systems without discarding social organization in the process. Notably, Coleman's framework suggests that an individual and social groups make rational choices (i.e individuals engage in social interactions, relationships and networks for as long as the benefits persist) in all phases of social life (29). These rational actions are set in a particular social context accounting for not only the actions of individuals, but also the development of social organization (Figure 1). The framework depicts a causal chain linking individual and organizational levels through intermediate steps. As such, accountability process contributes to observable performance, which are beneficial to many actors via relational accountability steps by a few actors. This is inspired by Coleman's concept of a ‘boat' linking macro-level conditions and outcomes via micro-level conditions (30).

The framework informed our study as iSAMs are created to guide members to reach a consensus and make decisions highly relevant to a particular context, more so where primary actors play a key role in supporting access to WASH services (31, 32). Further, it applies to our context where defined accountability mechanisms can bring forth desirable organizational outputs and outcomes (fulfilling WASH needs in childcare centres) through intermediate steps. Similar to Coleman's' ideas, the organizational outputs from co-creation of WASH service delivery will not only benefit the individual who were involved but also other actors in general. This is because social capital is both a private and public good benefiting everyone in the social group, not only those who invest in associations or networks. The notion that ‘direct contributions by some individuals will benefit the whole, not just the individual' could be an anticipated limitation for participation. However, in this study it enabled the researchers to sample dedicated participants, who willingly volunteered throughout the co-creation process.

## Methods

The study is reported per a set of standardized criteria for reporting qualitative research (COREQ) (33).

### Study objectives

Our study sought to document (a) co-creating iSAMs processes and (b) self-evaluation of the co-creation process for WASH service delivery in childcare centers within Nairobi's informal settlements.

### Study design

This was a qualitative study, using focus group discussions (FGDs) and stakeholder participatory workshops. Focus groups are semi-structured discussions with groups of 4–12 people that aim to explore a specific set of issues. Moderators often commence the focus group by asking broad questions about the topic of interest, before asking the focal questions. Participatory workshops entail a semi-structured discussion, with ~20–100 participants, who are deliberating on an issue and are usually complemented with FGDs. Although participants in FGDs and workshops get to individually answer the facilitator's questions, they are encouraged to talk and interact with each other. FGDs and workshops are built on the notion that group interaction encourages respondents to explore, reflect and clarify individual and shared perspectives, and adopts a collaborative participation pathways (33). Collaborative participatory pathways equitably involve community members, researchers, and other stakeholders in the research process, recognizing and maximizing the importance of their diverse contributions (34). In our study, the aim of collaborative participatory pathways through FGDs and workshops was to create a positive, transformative, and sustainability together with, for, and in communities. Additionally, collaboration enhanced a bottom-up approach to co-creation in WASH service delivery in childcare centers. This is because the process ensured a platform for many key actors to be heard and room for diversity, differences and desires.

### Study setting

**Figure 2:**
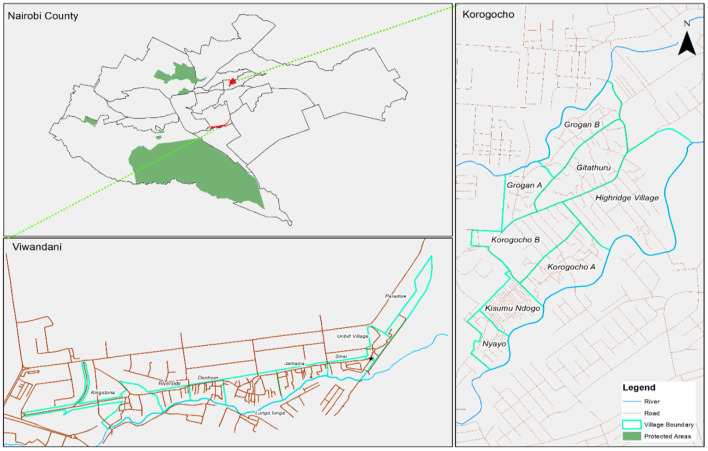
Study setting.

The study was conducted in Korogocho and Viwandani informal settlements in Nairobi, in the areas covered by Nairobi Urban Health and Demographic Surveillance System (NUHDSS) initiated in 2002 by the African Population and Health Research Center (APHRC) (35). Korogocho has a stable and settled population and residents have lived in the area for many years (36), while Viwandani is located next to an industrial area with many highly mobile residents who work or seek jobs in the industrial area (36). There are ~50 and 60 childcare centers in Korogocho and Viwandani, respectively with poor or no access to WASH services (32). ISAMs facilitated and enabled access to water, sanitation and hygiene services in childcare centers in both settlements (16). Each of the study sites is divided into 8 units/villages or polygons for ease with sampling (see Figure 2).

### Target population, sampling and sample size

The population of interest was childcare managers and parents with children under 5 years attending childcare centers. We sampled 100 participants comprising 34 childcare center owners catering to children under-5 years and 66 parents of children under-5 years attending the childcare centers in each of the two study sites. The participants took part in both the FGDs and the participatory workshops complemented with FGDs. Childcare center managers were selected if they were serving children in more than one of the 8 units in each of the study sites, this allowed a diversity of ideas. For each of the centers where the center managers were purposively recruited, at least two parents with a child in each of the centers were purposively selected (Table 1). We purposively selected parents/guardians who were key for child expenses in the family and who had children in childcare centers for the longest time compared to other parents. The length of stay was important as it portrays that they would likely participate in the study that entailed a series of participation.

**Table 1:** Sample size and demographic information.

**Characteristics (sex of participants)**	**Childcare managers (*n*)**	**Parents (*n*)**	**Total (*N*)**
**Korogocho (** ***n*** **)**			
Female	24	40	
Male	10	26	
**Total**	**34**	**66**	**100**
**Viwandani (** ***n*** **)**			
Female	24	50	
Male	10	16	
**Total**	**34**	**66**	**100**

### Data collection process

**Figure 3:**
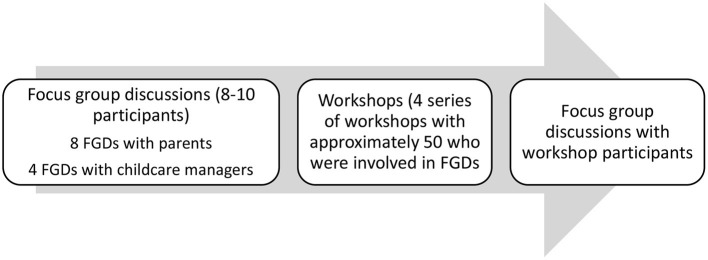
Data collection process.

We collected data in the two study sites from December 2021–May 2022 using FGDs and a workshop guide that had questions related to (a) co-creating iSAMs processes for WASH service delivery in childcare centers and (b) self-evaluation of the co-creation process. FGDs enabled the study participants to describe the steps to co-create the iSAMs process in the silo groups, while participatory workshops enabled the study participants to discuss and have a consensus on a combined co-created iSAMs process and to self-evaluate the process. The data collection process is further detailed and described in Figure 3.

At the formative stage, trained research assistants administered eight FGDs to parents of children attending childcare centers and four FGDs to childcare center managers in each of the two study sites. FGDs comprised a group of 8–10 participants and were mainly held at community halls identified by the study participants. Our data collection team comprised of a moderator, a note-taker and a team leader. The moderator guided the discussion, the note taker took notes and observed non-verbal cues, and the team leader's role was to oversee and troubleshoot any problems, clarify any issues or questions, consult with senior researchers, and perform spot checks to enhance the quality of data. The FGDs recorded sessions took ~45–60 mins.

Four series (two in each study site) of participatory workshops were held with ~50 study participants who were involved earlier in the FGDs. Each workshop was composed of approximate participants who were drawn from two groups of FGD members with childcare center owners and from four groups of FGD members with parents. The workshops took ~90 mins of recorded sessions. After each of the workshops, participants would sit in FGD sessions for ~45 mins to reflect and self-evaluate the iSAMs process. We collected data using audio recordings, flip charts, as well as workshop materials such as slides.

### Data quality control

Research Assistants were selected by researchers at the APHRC staff if they had at least 5 years of experience in qualitative research and were endorsed by the community in the study sites. The Research Assistants were trained for 5 days on the aims of the study, data collection process, data collection tools, facilitation of the co-creation process and research ethics. During fieldwork, field supervisors accompanied the research teams to ensure that probing was done correctly and to assess any threats to data quality. Debriefing sessions were held at the end of each working day to highlight the key findings, review probing techniques, and assess progress.

### Data analysis

The recordings from participatory workshops and FGDs were transcribed into MS Word and cross-checked by a third party to ensure that all the information had been captured in the transcript. The transcripts were translated from Swahili to English (where necessary) and again cross-checked to ensure that the translation did not alter the meanings of the data. Transcripts were imported into NVivo 12 software (QSR International, Australia) for coding and analysis. Each transcript had a unique identifier comprising of date, study site and sex of the participant to enhance anonymity and facilitate informed analysis.

We used a framework analysis (37), informed by Coleman's framework (29). Framework analysis is adopted for research that has specific questions, a pre-designed sample and priory issues (37). The first step of framework analysis was listening to the recordings to familiarize the researchers with the information related to co-creation processes and self-evaluation of co-creation. To ensure reliability, two researchers (an experienced qualitative researcher with WASH experience and an anthropologist) and five co-researchers, who collected the data participated in the development of a coding framework by reading the outputs imported in NVivo 12 software independently, to establish an inter-coder agreement. Once the initial coding framework was completed, the team met to discuss the themes generated and to reach an agreement on themes. Two researchers proceeded with coding, charting, mapping and interpretation of transcripts, guided by agreed themes and codes (Table 2). The themes and codes had two domains of process and evaluation.

**Table 2:** Codes and themes during analysis.

**1. The process of co-creating an informal social accountability mechanism**	
**Codes converted into themes**	
(a) Defining accountability to whom and for what?	
(b) Performing/ action and information	
(c) Judging and assessing performance	
(d) Learning and adapting	
(e) Key outcome-Met WASH needs of children	
**2. Self-evaluation of the co-creation process**	
**Major themes**	**Emerging themes**
1. Positive appraisal	(a) Improved understanding in childcare centers (b) Enhance inclusion and ownership (c) Tailoring of activities into relevant context (d) Improved performance and better outcomes
2. Negative appraisal	(a) Time constraints (b) Financial burdens (cost of implementing the process) (c) Few committed participants and leaders (d) Lack of consistent commitment to participate

### Ethical considerations

The study was conducted in accordance with the Declaration of Helsinki, and approved by AMREF Health Africa's Ethics and Scientific Review Committee (ESRC), REF: AMREF-ESRC P747/2020. We obtained a research permit from National Commission for Science, Technology and Innovation (NACOSTI), REF: NACOSTI/P/20/7726. Approval was also obtained from the Liverpool School of Tropical Medicine (LSTM) and the African Population and Health Research Centre (APHRC) internal ethical review committees. All participants provided informed written consent before participating in an interview including consent for using photos and videos if there were any.

## Results

In total 200 participants took part in our study, comprising childcare managers and parents (see Table 1) in the Methods section. We identified two domains (1) processes of co-creating iSAMs and (2) self-evaluation of iSAMs as summarized in Table 2.

### Co-creating an informal social accountability process

**Figure 4:**
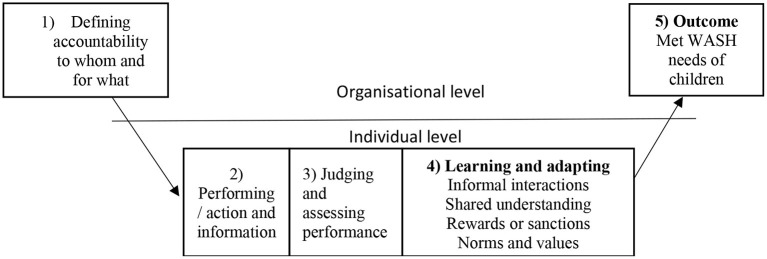
Co-creation model of informal social accountability process.

Data captured through FGD and workshops fed into the co-creating process. The iSAMs process co-created entailed four iterative steps that were proposed by participants to potentially improve WASH service delivery in childcare centers. The steps included (1) defining accountability, (2) action and information on social accountability actions based on definition(s), (3) making judgements and assessing performance about the appropriateness of the actions (affirming or imposing sanctions for unsatisfactory performance) and (4) learning and adapting based on judgements and assessment (Table 2). The four key steps identified by study participants were proposed to ultimately improve access to WASH services in childcare centers (see Figure 4).

#### Step one: Defining accountability to whom and for what

The study participants agreed that this was the first step of the co-creation model. Parents and childcare managers described the importance of the collaborative definition of accountability to whom and accountability for what? The study participants described the importance of accountability to children and to each other as the key actors in childcare centers, for access and utilization of WASH services and products by the children in childcare centers.

“*It is key to define accountability, for example, I am accountable to the child and the parent of the child because they are the reason why I am in this centre”* (Female Childcare center manager, Viwandani, 2022).

“*I am accountable and by the way, parents need to be accountable to childcare centre managers and the children as well. It is important to define and agree because some parents think that they are only accountable to their child”* (Female Parent, Korogocho, 2022).

In addition, parents felt the definition of accountability could enable parents to explore further on “accountability to whom” as such, parents thought they were accountable to not only childcare center managers and children but also to their neighbors. Childcare center managers also agreed on the same and described that they were also accountable to the government and local authorities.

“*When we define accountability to whom, we get to think further. As parents, we are also accountable to our neighbours. For example, if I do not ensure children have access to water, sanitation and hygiene at the centre, the children might not access, be sick and disturb the neighbours because we share sanitation facilities at home”* (Female Parent, Viwandani, 2022).

“*If we find a good time to define accountability to whom, it becomes an opportunity to think of actors we are accountable to. We are also accountable to the government and other authorities”* (Female Childcare center manager, Korogocho, 2022).

There were dynamics in the definition of accountability for what. Male parents described that there was no need to define accountability for what as they thought they would forget, while female parents embraced the need to define the same, as they thought it could catalyze accountability.

“*I think it is just ok to define accountability to whom; accountability for what is not necessary… this is because one can easily forget what they subscribed to and what they did not”* (Male Parent, Viwandani, 2022).

“*It is important to define accountability for what so that the centre manager can be keen on what you all agreed*” (Female Parent, Viwandani, 2022).

#### Step two: Action and information on social accountability actions

Study participants described how once accountability for WASH service delivery is defined, there is a need for the key actors (parents and childcare center managers) to act by ensuring the provision and utilization of WASH amenities by children. Parents described that their roles could include payment of childcare fees to enable childcare managers to purchase WASH amenities, or sometimes parents could purchase WASH amenities for children and deliver them to childcare centers. Parents expressed the willingness to prioritize availing the WASH needs of children in childcare centers, even when they lacked the amenities at their homes, as parents did not want to be interrupted by center managers at work for failing to avail the amenities.

“*Once the roles are defined, it is important to act by availing water, sanitation and hygiene amenities in childcare centres, payment of a fee for the childcare centre managers to purchase the amenities or by referring supporters to the centres”* (Female Parent, Viwandani, 2022).

“*I am ready to forgo having water in my house but ensure that the child access water and sanitation in the centre. This is because, I do not want to be interrupted by calls on the WASH needs by the centre managers, while I am at work*” (Male Parent, Viwandani, 2022).

“*I usually do not like my children to suffer when they are young. Anything we agree with the centre manager, I just avail; be it a fee or anything agreed. You know children spend most of their time there”* (Male Parent, Korogocho, 2022).

Center managers described the importance of availing WASH services and ensuring that children could utilize the amenities in the childcare centers.

“*It is important to avail water, sanitation and hygiene services and products in childcare centres for children to use”* (Male Childcare center manager, Viwandani, 2022).

“*Childcare managers should always act to ensure children use the water, sanitation and hygiene services and products at the center. There are times when I train children on the use of the WASH amenities”* (Female Childcare center manager, Korogocho, 2022).

Despite the willingness of many parents, study participants described that in some instances, some parents could not avail WASH services due to their inability to oblige to pay the fee or to avail the WASH amenities and products. This was reported to affect WASH service delivery.

”*There are some times when we are not able to avail adequate WASH amenities; more so when parents fail to take their responsibility of paying a fee or availing facilities at the centre”* (Male Childcare center manager, Korogocho, 2022).

“*Some parents are not able to avail WASH facilities or products in childcare centres or pay the fee. They affect the ability of childcare centre managers to provide the amenities in the centres”* (Female Parent, Viwandani, 2022).

#### Step three: Judging and assessing performance

Judging and assessing performance/action was thought to be key for continued provision and utilization of WASH amenities. As such, during action/performance, participants agreed on the importance of assessing the progress of action/performance.

“*Parents should monitor how children use WASH facilities and how Childcare managers are present to give guidance when needed. On the other hand Childcare managers should monitor how parents are responsible for availing WASH products for use in the centre.”* (Male Childcare center manager, Korogocho, 2022).

The assessment could potentially happen in many forms through visits to a childcare center.

“*It is always good to assess performance. While taking my child to school, I confirm if the centre has potty/toilet facilities and drinking water”* (Male Parent, Korogocho, 2022).

“*I find it important to visit my child in the centre at least once in a month to confirm if the child is being attended to and could utilise available water, sanitation and hygiene facilities”* (Female Parent, Viwandani, 2022).

“*I sent her older sister to go and check if the child could access water and sanitation facilities at the centre and if there is someone guiding the child”* (Male Parent, Korogocho, 2022).

Some parents, most of whom were male, were committed to other duties and did not visit the childcare centers. As such, the parents would do an assessment of WASH amenities and service delivery digitally hence, the need for the iSAMs process as a support to effective social accountability.

“*I usually ask the parents who take children to the centre on my behalf to find out about the status of WASH in the centre, where possible I ask them to take a photo of WASH facilities and share it with me*” (Male Parent, Korogocho, 2022).

“*In many cases, I ask my neighbour who constantly visits the centre to take some photos of the facilities and share them during her visits”* (Male Parent, Viwandani, 2022).

Judgement and assessment could also be done through reports.

“*I have to report to parents when they fail to meet what we agreed on regarding provision of WASH services… sometimes through a written note or phone calls”* (Female Childcare center manager, Viwandani, 2022).

“*As parents, we ask children to report on access to WASH facilities while at the centre. Although very young children could not report effectively*” (Male Parent, Korogocho, 2022).

#### Step four: Learning and adapting informal social accountability mechanisms

Study participants described how the assessment/judgement step led to learning and adapting informal social accountability mechanisms that included informal interactions, shared understanding, rewards, sanctions, norms and values among others; ISAMs identified in an early study (16).

“*If a centre manager is doing well, it is important to reward them as parents. The rewards are usually in form of availing free water or paying extra money for their good task … the rewards can be adapted over time to always be effective”* (Female Parent, Korogocho, 2022).

“*For parents who are not compliant with what we agreed, it is good to communicate with them, encourage and train them to pay in instalments for their children to access WASH facilities. However, when they cannot improve in their compliance, we do not allow them to bring their children to the centre”* (Female Childcare center manager, Viwandani, 2022).

#### Step five: Key outcome—Meeting the needs; WASH needs

Study participants described how learning and adapting informal social accountability mechanisms have the potential of enabling the actors to achieve the performance of WASH needs in childcare centers.

“*It is with the four steps that children could have access to water, sanitation and hygiene services in the childcare centres. This is because both parents and childcare centres are involved and responsible”* (Male Childcare center manager, Korogocho, 2022).

### Positive self-evaluation of the co-creation process

#### Improved understanding of childcare centers and building trust for WASH service delivery

Respondents mentioned that the discussions during the co-creation process resulted in new insights and awareness regarding childcare center operations, challenges, values and WASH service delivery.

“*One thing I take with me is that the childcare manager is investing in these matters {accountability for WASH service provision}. It is valuable to see that there is an ongoing concern. They take these matters seriously and engage with parents. So, it feels good”* (Female Parent, Viwandani, 2022).

Increased learning about accountability for WASH was mentioned, in that working with the researchers contributed to a more profound understanding of different aspects of social accountability and its relationship to WASH service delivery. The respondents described that, even though the concept of social accountability was not new to them, it was somewhat difficult to grasp and fully understand its practical meaning. The concept was operationalized and applied in their local context which then contributed to their greater understanding of the issues in focus. One participant whose views represented those of the majority described this in terms of getting a new, mutual and practical language.

“*So, the centre has gotten a lot out of this co-creation work. We as individuals have got a language, we had never used the word accountability before, but I have done so now. Yes, so we have learned and gotten a lot out of this, the language seems practical to us”* (Female Childcare center manager, Korogocho, 2022).

#### Enhance inclusion, ownership and a satisfying co-creation process

Study participants acknowledged how the co-creation process was mutual and both parents and center managers shared responsibilities. The participants noted that there was a great engagement of all during the workshop, a good grounding in the concepts, and informative with a great diversity in the participants. As such, the respondents expressed that they were satisfied with the balanced responsibilities and ownership throughout the whole process.

“*We have tried to take responsibility and we have felt a sense of inclusion and ownership in the process, therefore, we are satisfied with the entire process, we deliberated a lot”* (Female Childcare center manager, Viwandani, 2022).

Many respondents expressed satisfaction with the collaboration in the overall co-creation structure as there were representatives of different primary actors. The participants perceived the co-creation process as straightforward, and a helpful tool to identify outcomes and processes. Some respondents desired to try the model for other service delivery challenges other than WASH.

“*We do not have everyone involved but as representatives, it becomes easier for us to convince others. I will convince other parents. Childcare managers who are here will also convince their colleagues to apply the process to WASH service delivery and other services”* (Male Parent, Viwandani, 2022).

Time, date and other plans of conducting FGDs and workshops were participants-led, as such, respondents expressed satisfaction in the process. Several respondents also expressed contentment with being listened to and said that they had the opportunity to have their voices heard.

“*We were allowed to choose the best time to attend the session and we committed to the sessions. I think the researchers listened to us like never before. Which was helpful to all of us”* (Female Childcare center manager, Viwandani, 2022).

#### Tailoring of activities and strategies into relevant context

Study participants described how Involving childcare managers and parents during the co-creation of iSAMs enabled a good contextual fit, as the activities were relevant to the context. The participants also termed the activities to have enhanced willingness to implement the process. This could be seen as an example of tailoring activities and processes, not only for improved WASH service delivery but also to simultaneously contribute to the overall performance of a childcare center.

“*I think that the processes are relevant to us; activities are fully relevant, and I think it will lead to good accountability for WASH service delivery and other outputs in the centre”* (Male Childcare center manager, Viwandani, 2022).

“*The process leads to learning from each other directly and can bring so much value to WASH service delivery in childcare centres”* (Male Parent, Korogocho, 2022).

#### Potential for improved performance and better outcomes

While self-appraising the use of co-creation between the childcare managers and the parents regarding WASH service provision, it was unanimously agreed that co-creation had the potential to enhance WASH service delivery outcomes. Childcare managers and parents engaged in this process as they believed it would improve WASH service delivery in childcare centers.

“*In due course, with co-creation of social accountability mechanisms process by parents and childcare centres in WASH service delivery, it is likely that children may have better WASH services”* (Female Childcare center manager, Korogocho, 2022).

“*It's been a great partnership, with true successes. The process has enabled ownership of approaches and sharing of skill sets. We incorporated some activities during this period and are in the process of incorporating others going forward”* (Female Parent, Viwandani, 2022).

### Negative self-evaluation of the co-creation process

Study participants identified various challenges to effective co-creation. These challenges were around timing and organization. Further, the participants described how it could be challenging to consistently have committed local contributors and committed leaders.

“*Amount of time that goes into the co-creation process can be challenging… the entire steps consumes a lot of time”* (Male Parent, Viwandani, 2022).

“*I needed to be at a community forum but I had to give an apology so that I could join this meeting”* (Female Childcare center manager, Viwandani, 2022).

“*Lots of people usually want to contribute liberally, but I do feel it needs some strong leadership from all actors involved”* (Male Parent, Korogocho, 2022).

A final challenge was a need to ensure effective co-creation and positive outcomes, with limited resources and planning, while taking cognizant of social, structural and economic challenges of the local context in the informal settlements. As such, most parents and childcare center owners who were underprivileged compared to the ones who were privileged raised this concern. For instance, some parents reported a lack of finance to purchase WASH facilities for their children. Several of the childcare center owners and parents were positive but pointed out a lack of consistent commitment by some actors to perform their roles.

“*One challenge is the lack of consistent commitment of partners and stakeholders to do the needful that will promote co-creation”* (Female Childcare center manager, Korogocho, 2022).

“*The co-creation process was good but sometimes; we have inadequate resources and cannot afford to purchase potties, soap, water or buckets for hand washing”* (Female Parent, Viwandani, 2022).

## Discussion

Sustainable Development Goal (SDG) target 17 implicitly recommends a co-creation approach to achieving sustainability in service delivery because it emphasizes the central role of partnerships, equally, target 6.b focuses on a need for participation of local communities in water and sanitation planning and management (38). Partnerships among researchers, parents and childcare managers during co-creation served as “force multipliers” in generating collaborative ideas that could lead to sustainable solutions. Co-creation has a potential to mobilize collective energy, harness distributed knowledge and resources, engage in processes of mutual learning, develop prototypes and implement new and bold solutions that can be jointly evaluated and improved (38). By drawing together varied perspectives of actors, co-creation fosters nuanced problem understandings and mobilizes the local knowledge and other resources crucial for context-sensitive local solutions. This study explored the co-creation process of iSAMs for WASH service delivery in childcare centers in two informal settlements in Nairobi, Kenya, and the self-evaluation of the process using FGDs and workshops. The co-creation process in our study involved collaboration and contributions by childcare managers and parents, who are key actors in WASH service delivery in childcare centers in informal settlements (32). Co-creation depends on increased cooperation as well as strong partnership processes between different stakeholders. Notably, the study participants study participants identified (1) four steps of co-creating iSAMs resulting in an output and (2) positive and negative appraisals of the process in access to WASH services by children in childcare centers. Further interrogation and analysis of the iSAMs process and self-evaluation in the two study sites did not show any difference.

Co-creation processes in other contexts describe iterative processes, which are closely related to our findings. For example, a co-creation process that involved employees defined four building blocks of the process: (1) providing structure in the creation process, (2) implementing motivational elements, (3) creating emotional proximity and ownership, and (4) offering feedback on learning material for quality assurance (22). The co-creation process however involved one set of actors (employees). Our study will add to the literature as both the users (parents) and providers (childcare managers) were involved in describing the building blocks of co-creation using two qualitative approaches; FGDs and workshops. The co-creation process output entailed (1) defining accountability to whom and for what?, (2) performing/action and information, (3) judging and assessing performance, (4) learning and adapting, the four steps were intended to lead to (5) a key outcome of meeting WASH needs of children in childcare centers. It was clear that FGDs and participatory workshop approaches for co-creating iSAMs were of great value, as the participants were beneficiaries of the output and had attribution in the process. Consequently, we grounded this work on Coleman's framework with a focus on social capital and stressing the role that individuals play in an organization (29). The framework was key in planning and development of the iterative process. This is because the organizational outputs from co-creation of WASH service delivery will not only benefit individuals who were involved but also other actors in general. For example, an improved WASH service delivery output as a result of co-creation will benefit all children in childcare centres, including children of parents who were not involved in the process.

Positive appraisal of the co-creation process included equal involvement and collaboration of study participants. Collaborative influence over the process was linked to enhanced WASH service delivery in childcare centers. In preceding studies, the need to co-create strategies to access WASH services in public spaces including childcare centers have been stressed to be important (27, 28), to counter the narrative that collaboration of explicit strategies is often left out in participatory work environment (21, 38). This implies that the process fostered a good fit into the context, a gap that was identified in the introduction section. The use of co-creation by researchers, end-users, and other relevant stakeholders when developing interventions is increasingly encouraged (21). However, few studies have described a co-creation process and self-evaluation of the process or more distal outcomes such as improving access to WASH needs among end-users (22, 28). As such, to our knowledge, this is the first study documenting the co-creation process and self-evaluation of the process in WASH service delivery in childcare centers, so as to fill in the gap. This study adds to practice by documenting the co-creation process and self-evaluation of the process that can be adopted by actors for improved outcomes. As mentioned in the introduction, defining an implementation strategy and involving end-users in co-creation appears rare (19, 21). An insight from our findings is that end-users can be involved in the co-creation process and can provide a distinction between the process and the outcome. Despite the positive appraisals, there were negative appraisals that included the ongoing collaborative nature of co-creation which is time-consuming and requires a lot of organizational skills for a meaningful and successful deliverable. The balance of who and how many people to involve, to ensure progression yet still ensure an inclusive process, alongside ensuring the process is driven forward by somebody taking the lead role as noted by our study participants can pose a challenge, as can the selection of less motivated partners. These negative appraisals and concerns do not negate the very positive effects of co-creation, but need identifying at an early stage while planning on co-creation approaches.

Our findings imply that sustainable and equitable WASH service delivery in childcare centers in informal settlements needs research that goes beyond raising awareness to fully engage and collaborate to ensure that novel solutions are developed at an appropriate scale to meet specific needs (21, 25). Applying co-creation can be a valuable method for adopting iSAMs and facilitating service delivery. As such, there is a need to integrate co-creation in already existing structures in childcare centers (16). Co-creation process can identify multiple solutions to WASH service delivery, which can be adapted and tailored to childcare centers.

## Strengths and limitations

Our strengths included strong networks in the study sites, well-trained and skilled data collectors recruited from the community and the ability to use an existing framework for analysis. This heightened our drive towards the validity of the study results. Our study is not without limitations. This study was conducted in only two informal settlements in Nairobi with key stakeholders in childcare centers. The findings were necessary for exploring the co-creation process and self-evaluation of co-creation for WASH service delivery in childcare centers. Nonetheless, a more holistic approach that combines qualitative and quantitative data, and integrates more stakeholders would be necessary for a broader understanding of the many aspects of the study, moving forward.

## Conclusion

First during the co-creating process, actors realize their potential during initial engagement meetings (i.e., that they are not only capable of producing innovative, yet feasible solutions, but they also help to build broad-based ownership to new and bold solutions), thus enhancing democratic legitimacy. Second, collaborative interaction in co-creation arenas tends to empower the participating actors and build resilient communities that are capable of bouncing back when facing stress, turbulence or disruptive crises. Third, the co-created process identified in our study seems to be feasible in childcare centers elsewhere in Kenya and other LMIC settings. This is because the process reduces contextual barriers which are often overlooked, as it allows understanding of issues through the “eyes” of people who experience service delivery issues. Consequently, other users and service providers could adopt the co-creation process and streamline the same for quality service delivery in the WASH sector and other sectors. Fourth, prioritizing co-creation can effectively identify tailored approaches to strengthen WASH service delivery. These approaches could provide a model to guide future local participatory action research for improving WASH service delivery and other basic services in Kenya's informal settlements and other under-resourced settings. Fifth, our co-creation process model could be further enhanced by a “champion” or an “implementation team” for sustainability. The process can form a locally tailored model, which encourages the engagement of more vulnerable members and disadvantaged groups, leading to improved outputs across communities. Ensuring more actors have an equitable seat at the table can contribute to the good governance needed to strengthen WASH systems and achieve SDG 6 targets for water and sanitation. To support this, there is potential for key actors to focus on “quick win” solutions, as it offers insight and recommendations for co-designing, co-production and co-creation of local knowledge and practical solutions that could be scaled up to operationalize social accountability more widely.

Lastly, situational leadership aiming to diagnose problems and to try out different solutions is called for and will often trump any list of recommendations, more so in informal settlements, where service delivery is dominated by informality. Despite that many local change makers might not get as far as initiating, leading and managing co-creation processes in informal settlements, and that many governments, economic elites and local power-holders may suppress social entrepreneurship and unsolicited social action. Fortunately, in Kenya, government actors and elites welcome bottom-up initiatives, similar to our process that could help to solve urgent problems and achieve important sustainability goals such as those captured by the SDGs, including partnerships in WASH service delivery in informal settlements. Overall, sustainable and equitable WASH service delivery in childcare centres in informal settlements needs research that goes beyond raising awareness to fully engage and co-create to ensure that novel solutions are developed at an appropriate scale to meet specific needs. We recommend that actors should integrate co-created approaches in already existing structures for water, sanitation and hygiene service delivery in childcare centers and in other contexts. Future research should aim to understand factors that promote the integration and sustainability of functional social accountability processes aimed at improving the WASH services.

## Plain English

Parents and childcare managers play a key role in provision of water, sanitation and hygiene for use by children in childcare centers in informal settlements. There are approaches referred to as “informal social accountability mechanism” used by both parents and center managers to hold each other responsible for service delivery. To date, the approaches have shown a limited effect, often due to implementation failures and poor contextual fit. As such, we explored the approaches and documented the process and appraisals by involving both parents and childcare managers. We identified a four-stepped process that led to an outcome and both positive and negative appraisals of the process. We conclude that sustainable water, sanitation and hygiene service delivery in childcare centers in informal settlements needs research that goes beyond raising awareness to fully engage and co-create feasible novel solutions to meet specific water, sanitation and hygiene needs. We recommend that actors should integrate co-created approaches in already existing structures for water, sanitation and hygiene service delivery in childcare centers and in other contexts.

## Data availability statement

The raw data supporting the conclusions of this article will be made available by the authors, without undue reservation.

## Ethics statement

The study was approved by AMREF Health Africa's Ethics and Scientific Review Committee (ESRC), REF: AMREF-ESRC P747/2020, and a reserch permit from National Commission for Science, Technology and Innovation (NACOSTI), REF: NACOSTI/P/20/7726. Approval was also obtained from the Liverpool School of Tropical Medicine (LSTM) and the African Population and Health Research Centre (APHRC) internal ethical review committees. All participants provided informed written consent before participating in an interview including consent for using photos and videos if there were any. The patients/participants provided their written informed consent to participate in this study.

## Author contributions

Conceptualization, data curation and analysis, methodology, project administration, and first draft: IC. Validation: IC, CK, HE, PP-H, and BM. Review and editing: IC, CK, HE, KO, PP-H, and BM. Supervision: PP-H, HE, and BM. All authors approved the manuscript for submission.
